# Efficacy of non-opioid analgesics to control postoperative pain: a network meta-analysis

**DOI:** 10.1186/s12871-020-01147-y

**Published:** 2020-10-27

**Authors:** John A. Carter, Libby K. Black, Dolly Sharma, Tarun Bhagnani, Jonathan S. Jahr

**Affiliations:** 1 Blue Point LLC, 711 Warrenville Road, Wheaton, IL 60189 USA; 2 Baudax Bio Inc, Malvern, PA USA; 3 EPI-Q, Inc, Oak Brook, IL USA; 4 Department of Anesthesiology and Perioperative Medicine, UCLA, Los Angeles, CA USA

**Keywords:** Analgesia, Meloxicam, Opioid, Pain assessment, Postoperative Pain

## Abstract

**Background:**

The aim of this network meta-analysis (NMA) was to evaluate the safety and efficacy of intravenous (IV) Meloxicam 30 mg (MIV), an investigational non-steroidal anti-inflammatory drug (NSAID), and certain other IV non-opioid analgesics for moderate-severe acute postoperative pain.

**Methods:**

We searched PubMed and CENTRAL for Randomized Controlled Trials (RCT) (years 2000–2019, adult human subjects) of IV non-opioid analgesics (IV NSAIDs or IV Acetaminophen) used to treat acute pain after abdominal, hysterectomy, bunionectomy or orthopedic procedures. A Bayesian NMA was conducted in R to rank treatments based on the standardized mean differences in sum of pain intensity difference from baseline up to 24 h postoperatively (sum of pain intensity difference: SPID 24). The probability and the cumulative probability of rank for each treatment were calculated, and the surface under the cumulative ranking curve (SUCRA) was applied to distinguish treatments on the basis of their outcomes such that higher SUCRA values indicate better outcomes. The study protocol was prospectively registered with by PROSPERO (CRD42019117360).

**Results:**

Out of 2313 screened studies, 27 studies with 36 comparative observations were included, producing a treatment network that included the four non-opioid IV pain medications of interest (MIV, ketorolac, acetaminophen, and ibuprofen). MIV was associated with the largest SPID 24 for all procedure categories and comparators. The SUCRA ranking table indicated that MIV had the highest probability for the most effective treatment for abdominal (89.5%), bunionectomy (100%), and hysterectomy (99.8%). MIV was associated with significantly less MME utilization versus all comparators for abdominal procedures, hysterectomy, and versus acetaminophen in orthopedic procedures. Elsewhere MME utilization outcomes for MIV were largely equivalent or nominally better than other comparators. Odds of ORADEs were significantly higher for all comparators vs MIV for orthopedic (gastrointestinal) and hysterectomy (respiratory).

**Conclusions:**

MIV 30 mg may provide better pain reduction with similar or better safety compared to other approved IV non-opioid analgesics. Caution is warranted in interpreting these results as all comparisons involving MIV were indirect.

## Background

Management of postoperative pain remains a significant issue, including providing adequate pain control beyond immediate postsurgical recovery [1–3]. Poorly managed acute postoperative pain may have a significant impact on clinical and economic outcomes and is a consistent risk factor for persistent or chronic postoperative pain [3–5]. Opioid analgesics are the foundation of treatment for moderate-to-severe postsurgical pain and are among the most effective agents for the management of pain in many settings [6]. However, opioids are associated with the potential risks of opioid-related adverse drug events (ORADEs), (such as respiratory and gastrointestinal related events) and abuse or dependence, which can significantly increase the cost of medical care [7–9].

Multimodal pain management guidelines have been developed that provide guidance on reducing opioid monotherapy and the doses of opioids used to treat acute pain, while still providing effective pain management [10–12] This approach involves the administration of various opioid and non-opioid agents that act on different sites, resulting in a synergistic and additive effect [10–12]. The goal of multimodal pain management is to reduce ORADEs and their costs, as well as the risks of opioid abuse or dependence [13].

Non-opioid pharmacologic therapies for potential use in the multimodal regimen include acetaminophen and/or non-steroidal anti-inflammatory drugs (NSAIDs). Recent practice guidelines have recommended that unless contraindicated, all patients should receive around-the-clock treatment with acetaminophen or NSAIDs as part of multimodal analgesia for post-operative pain management [14]. When NSAIDs and/or acetaminophen are included in treatment regimens with opioids for pain relief, an opioid-sparing effect has been demonstrated [15]. Intravenous use of NSAIDs can achieve a faster onset of action and peak plasma concentrations compared to oral treatment regimens [10]. Parenteral formulations of ketorolac and ibuprofen were the only IV NSAIDs currently approved for postoperative pain management in the United States (US) at the time this study was conducted [16]. Studies have found that ketorolac reduces opioid consumption by 25–45% and provides additional benefits such as improving bowel function after colorectal surgery and epidural pain after cesarean delivery [17–19]. Intravenous ibuprofen is approved for the management of mild to moderate pain and for the management of moderate to severe pain as an adjunct to opioid analgesics [20]. Another non-opioid analgesic, acetaminophen, has an onset of action of 15 min when given as IV which is faster than the oral formulation and is associated with opioid-sparing effects [21].

NSAIDs act by inhibiting prostaglandin production through acetylation of cyclooxygenase (COX-1 and/or COX-2). Most NSAIDs are non-selective (i.e. they inhibit the activity of both COX-1 and COX-2). Inhibition of COX-1 activity is considered as a major contributor to NSAID gastrointestinal toxicity [22]. Non-selective NSAIDs are associated with an increased risk of gastrointestinal bleeding, cardiotoxicity, hepatotoxicity, renal dysfunction, and drug induced asthma [20, 23]. Ketorolac, the most widely used IV NSAIDs, has demonstrated a higher risk of gastro-toxicity and gastroduodenal lesions [24]. NSAIDs that selectively inhibit COX-2 are associated with fewer gastrointestinal effects [25, 26]. However, studies have linked selective COX-2 inhibitor to higher risk of myocardial infarction, stroke, and death [27].

A formulation of intravenous meloxicam (meloxicam IV; Anjeso™) (MIV) that utilizes a novel nanocrystal formulation has been approved by the U.S. Food and Drug Administration for the management of moderate-to-severe pain alone or in combination with other analgesics [28]. It belongs to the oxicam family of chemicals and blocks COX-2 more than it does COX-1, thus having fewer gastrointestinal side effects compared to non-selective NSAIDs, and without interfering with platelet function [29, 30]. Its efficacy and safety have been evaluated in seven Phase 2/3 randomized controlled clinical trials (RCTs) following procedures including dental surgery, abdominal hysterectomy, bunionectomy, abdominoplasty, and other major procedures [31–37]. Since these trials did not allow concomitant NSAID use and were placebo controlled, MIV has not yet been compared to other non-opioid IV analgesics. Hence, we conducted a network-meta-analysis (NMA) to assess the safety and efficacy of MIV relative to other IV non-opioid analgesics for moderate-severe postoperative pain. The study was conducted according to the Preferred Reporting Items for Systematic Reviews and Meta-Analyses (PRISMA) guidelines, Cochrane Handbook for Systematic Review of Interventions, and the International Society for Pharmacoeconomics and Outcomes Research (ISPOR) task force on Indirect Comparisons and Good Research Practices.

## Methods

### Search strategy

Using a pre-specified protocol, which was registered with PROSPERO (CRD42019117360), a systematic search was conducted in PubMed, Medline, EBSCO, Web of Science, Scopus, ClinicalTrials.gov, and Cochrane CENTRAL to identify randomized clinical trials from 2000 to 2019 and involving at least one of the following procedures or procedure groups: open abdominal (excluding hysterectomy), bunionectomy, open hysterectomy, orthopedic (joint replacement including knee, ankle, hip, shoulder). The literature search included publications on RCTs that reported clinical effectiveness, tolerability/safety, in adult patients receiving post-operative pain treatment. The search had no limits with respect to language or country.

### Study selection and eligibility criteria

In the first round of screening, all titles and abstracts were screened by a single investigator against the inclusion and exclusion criteria, using the PICOT criteria (population, interventions, comparators, outcomes, time period). The inclusion criteria for this NMA were: Studies that were conducted between 2000 and 2019 and that were RCTs; studies with adult patients (≥ 18 years) treated for post-operative pain involving one of the following procedures including, open abdominal (excluding hysterectomy), bunionectomy, open hysterectomy, orthopedic (joint replacement including knee, ankle, hip, shoulder); post-operative treatment with at least one non-opioid pain medication; and studies with the outcomes of ORADEs, opioid utilization, and pain intensity. Studies were eligible only if they included these comparators in at least one treatment group administered as follows: product was not administered continuously or as an infiltration, patients were randomized to product postoperatively in response to objectively measured moderate-severe pain (i.e., no preemptive administration), follow-up was conducted ≥12 h postoperatively.

A senior investigator validated 10% of the rejected abstracts to confirm accuracy. Abstracts with insufficient information were included. Full-text articles for the included abstracts were retrieved for in-depth review in the second round of screening, conducted by a single investigator using the same inclusion and exclusion criteria applied at the abstract level. A second investigator confirmed all excluded studies; any discrepancies were resolved by both the investigators together. Throughout the process, discrepancies were addressed by consensus between the two investigators. All screenings, extractions and validations were conducted in a shared Covidence database.

Two types of study selection criteria, restrictive and broad, were used to conduct this NMA. Under the restrictive study selection criteria, the studies were required to have waited until patients reached moderate-severe pain before they were administered the study analgesic. No such criteria were used for the broader analysis. The current study focuses on the results from the restrictive analysis as it better aligns with the clinical conditions in which MIV has been evaluated (i.e., postoperative moderate-severe pain). Results from the broader analysis are not reported here but may be available upon request.

### Data extraction

Full data extraction was performed on all studies included following the second round of screening. Extracted data included study descriptors, patient characteristics, treatment-level information, and outcomes (pain intensity, ORADEs). Data was extracted by two independent reviewers. Any discrepancies were resolved by agreement and consensus of the two investigators. Adjudication and extractions were made in a shared Covidence database (data held in a commonly shared Review Manager database). Where not reported, original confidence intervals were imputed based on information from the study reporting the point estimate combined with information from the literature regarding variability in the given endpoints.

### Outcomes and statistical analysis

Bayesian NMA was conducted using the netmeta and GeMTC packages in R to pool effect sizes of direct and indirect comparisons. The main outcomes analyzed were sum of pain intensity difference (SPID), total morphine milligram equivalents (MME) used, and ORADE frequency. SPID 24 (i.e., up to 24 h postoperatively) was chosen as the target pain outcome because it was expected that reporting beyond this timeframe would not be consistent across studies and we required a common timeframe to make comparisons across procedure groups. Two types of ORADEs were included in the analysis: respiratory (e.g. pulmonary congestion & hypostasis, pulmonary insufficiency following surgery and trauma, respiratory complications, other pulmonary insufficiency, bradypnea, acute respiratory failure, hypoxemia, hypoxia, mechanical ventilator) and gastrointestinal (e.g., paralytic ileus, postoperative ileus, constipation, nausea/vomiting). Sample-weighted mean differences were used for data measured on the same scale, and standardized mean differences (SMDs) were used where scales were not the same. Continuous outcomes (i.e., SPID and MMEs) were evaluated as mean differences versus placebo and dichotomous outcomes (i.e., ORADEs) as odds ratios (ORs). Where not otherwise reported, standard deviations were imputed using methods specified in the Cochrane guidelines (Section 16.1.3.2) [38]. A fixed effect approach was chosen for pain and MME outcomes due to the homogeneity of the study designs for those outcomes at the procedure level. Mixed-effects was used for ORADE outcomes given the heterogeneous compositions of the constituent adverse event categories [39].

Markov chain Monte Carlo methods were used to derive 95% credible intervals (CrIs). Credible intervals of the posterior distribution represented estimates for effect sizes, which can be interpreted similarly to confidence intervals [40]. The probability and the cumulative probability of rank for each treatment were calculated, and the surface under the cumulative ranking curve (SUCRA) was applied to distinguish each treatment by efficacy and safety where higher SUCRA values indicated better outcomes.

## Results

### Study selection

**Fig. 1:**
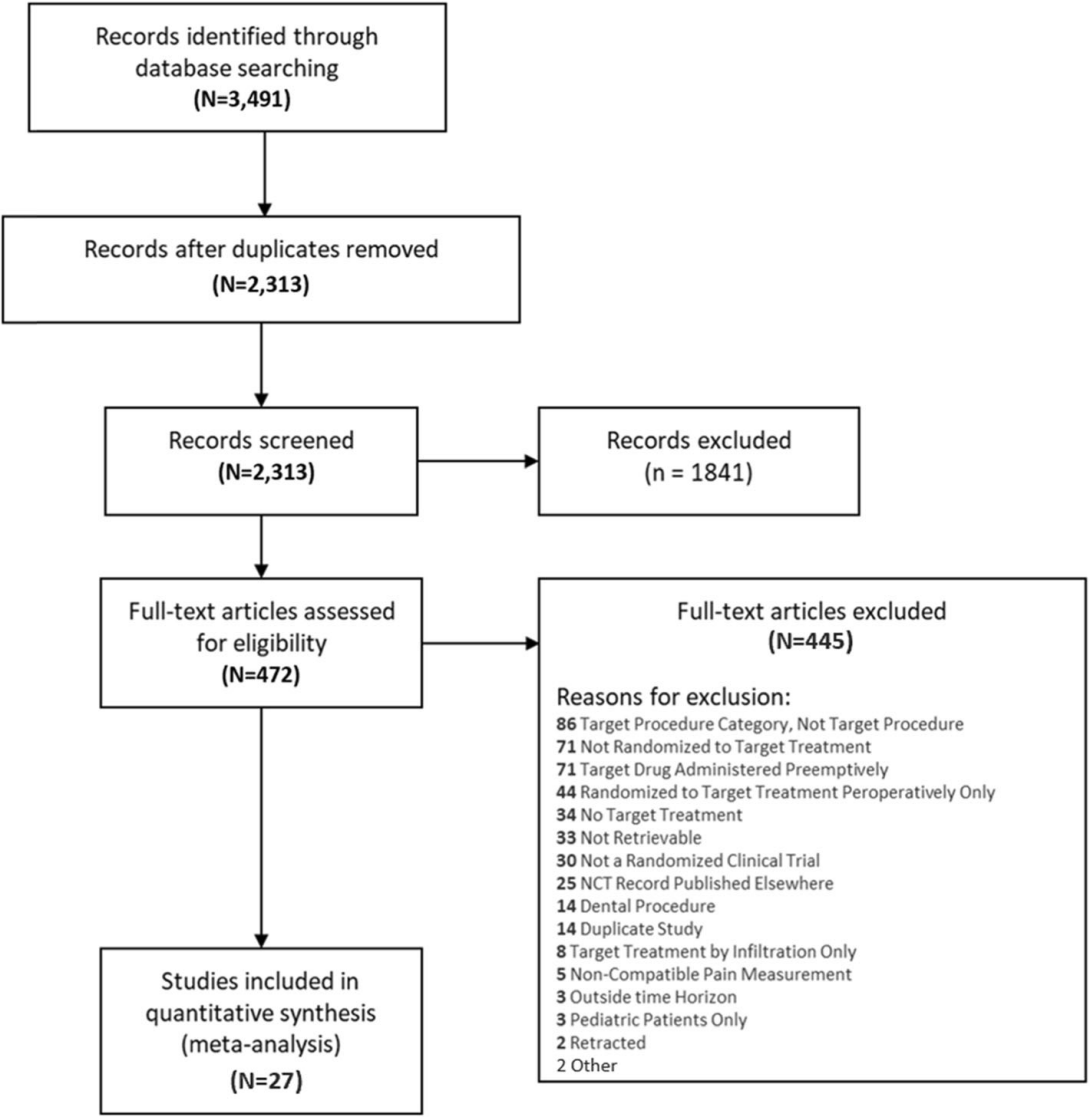
PRISMA flow diagram for record adjudication

A total of 2313 unduplicated study abstracts identified through literature search were screened for eligibility. Full text articles of 472 abstracts that met the inclusion/exclusion criteria were further screened, to identify 27 RCTs included in the analysis (Fig. 1). Eighty-six of the 445 excluded full-text studies were used in various capacities for generating informative priors. Informative priors were used to dictate the appropriate probability distributions for the Bayesian analysis.

**Fig. 2:**
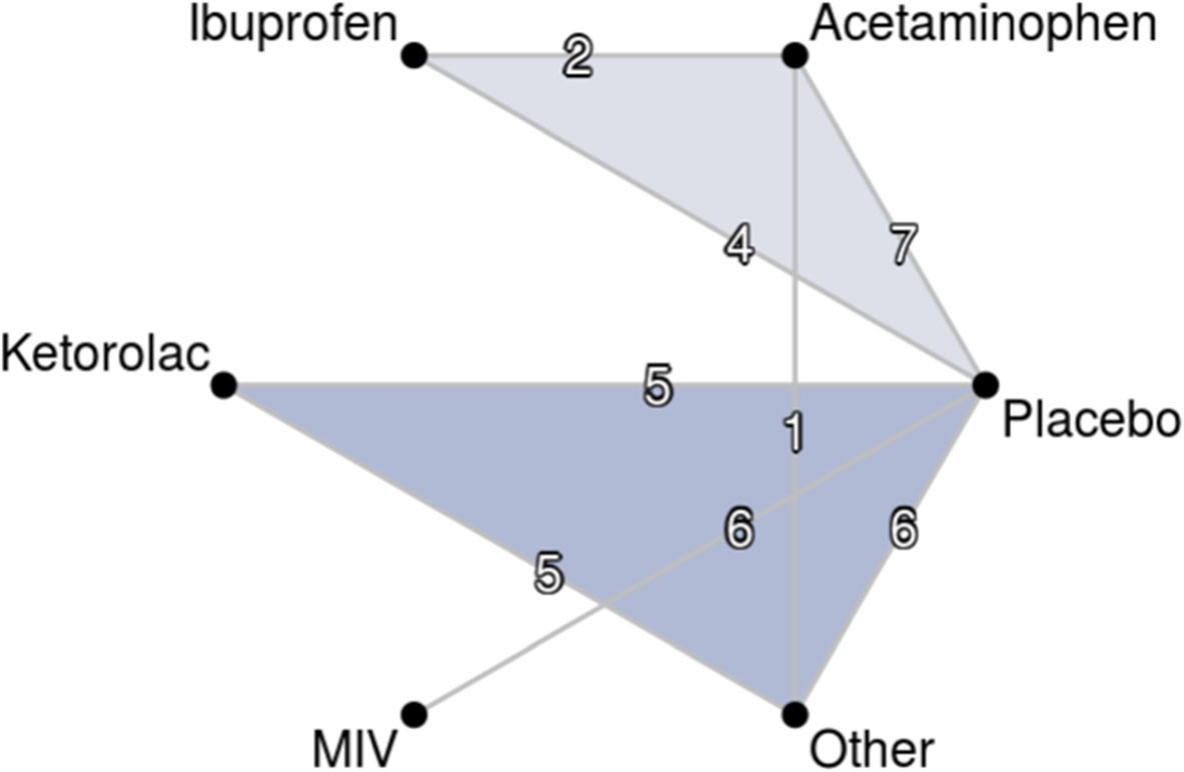
Network of 36 observations from 27 clinical trials for the primary outcome (SPID)

The characteristics of the 27 included studies are described in Table 1 [32–36, 41–62]. The network evaluated for all drugs and procedure types in this study is shown in Fig. 2. MIV was indirectly compared with only those IV treatments that were available at the time in the US (acetaminophen, ibuprofen and ketorolac). Studies for other non-opioid analgesics such as parecoxib and diclofenac were used for indirect comparison with the placebo arms in those studies.

**Table 1:** Characteristics of the RCTs included in this study (*N* = 27)

Author, Year (Procedure)	Sample Size	Treatments	Pain		MMEs Consumed				ORADEs		
			Included	SPID	Included	Hour 24	Hour 48	Hour 72	Included	GI	Respiratory/ Cardiovascular
Apfelbaum 2008 [41] (Bunionectomy)	255	Parecoxib (60 mg)	Yes	−50.64	No	–	–	–	Yes	0.21	–
		Parecoxib (40 mg)		−62.69		–	–	–		0.24	–
		Placebo		−65.22		–	–	–		0.38	–
Bakhsha 2016 [42] (Cesarean)	60	Diclofenac (suppository)^A^ / Placebo	Yes	− 19.26	No	–	–	–	No	–	–
		Acetaminophen		−21.01		–	–	–		–	–
Bangash 2018 [43] (Multiple, Elective)	220	Ketorolac + Acetaminophen	Yes	−47.95	No	–	–	–	No	–	–
		Ketorolac + Placebo		−41.74		–	–	–		–	–
Bergese 2017 [36]^B^ (Multiple)	720	MIV	No	–	Yes	–	26.3	28.4	Yes	0.39	0.00
		Placebo		–		–	34.3	37.4		0.49	0.00
Berkowitz 2017 [44] (Orthopedic)^B^	379	MIV	No	–	Yes	22.1	33.5	35.45	Yes	0.43	0.01
		Placebo		–		31.1	46.3	47.84		1.34	0.02
Bikhazi 2004 [45] (Hysterectomy)	329	Parecoxib (60 mg)	Yes	−18.2	No	–	–	–	Yes	0.71	–
		Parecoxib (40 mg)		−71.04		–	–	–		0.61	–
		Ketorolac (30 mg)		−91.28		–	–	–		0.55	–
		Placebo		−14.8		–	–	–		0.44	–
		Morphine (4 mg)		−45.24		–	–	–		0.66	–
Castro 2000 [46] (Abdominal)	230	Tramadol (100 mg)	Yes	−31.73	No	–	–	–	No	–	–
		Metamizol (2000 mg)		−58.93		–	–	–		–	–
		Ketorolac (30 mg)		−3.08		–	–	–		–	–
Daniels 2019 [47] (Bunionectomy)	276	Acetaminophen	Yes	−9.6	Yes^E^	–	45.00	–	No	–	–
		Ibuprofen		−8.6		–	37.50	–		–	–
		Placebo		1.5		–	61.50	–		–	–
Daniels 2013 [48] (Orthopedic)	277	Diclofenac	No	–	Yes	27.96	33.49	35.42	Yes	0.46	–
		Ketorolac (15–30 mg)		–		34.41	46.27	53.97		0.52	–
		Placebo		–		47.82	56.94	61.27		0.71	–
Essex 2018 [49] (Orthopedic)	116	Acetaminophen	No	–	No	–	–	–	Yes	–	0.02
		Placebo		–		–	–	–		–	0.07
Gago Martinez 2016 [50] (Abdominal)^D^	135	Ibuprofen	Yes	−24.5	Yes	17.36	26.32	–	No	–	–
		Placebo		−18.11		32.18	38.53	–		–	–
Gan 2012 [51] (Abdominal)	132	Placebo	Yes	−17.64	Yes	11.20	15.60	15.90	Yes	0.96	0.07
		Ketorolac (30 mg)		−27.56		6.70	8.53	8.50		0.79	0.05
		Diclofenac (18,75 mg)		−24.4		6.80	8.54	8.80		0.86	0.02
		Diclofenac (37.5 mg)		−61.08		–	–	–		–	–
Gottlieb 2018 [33] (Bunionectomy)^B^	96	MIV	Yes	−21.4	Yes^E^	–	57.40	–	No	–	–
		Placebo		10.32		–	77.00	–		–	–
Hynes 2006 [52] (Orthopedic)	120	Acetaminophen	No	–	No	–	–	–	Yes	0.05	0.00
		Diclofenac		–		–	–	–		0.05	0.075
Kim 2001 [53] (Abdominal)^D^	22	Ketorolac	Yes	−33.71	Yes	10.00	20.67	–	No	–	–
		Placebo		−26.95		28.00	34.88	–		–	–
Kroll 2010 [54] (Hysterectomy)^D^	319	Ibuprofen (800 mg)	Yes	−26.82	Yes	47.3	71.72	–	Yes	0.64	0.04
		Placebo		−20.09		55.9	66.92	–		0.70	0.02
Pareek 2011 [55] (Orthopedic)	158	Etodolac	No	–	No	–	–	–	Yes	0.05	–
		Diclofenac		–		–	–	–		0.05	–
Pollak 2018 [35] (Bunionectomy)^B^	120	MIV	Yes	−50.4	Yes	–	27.25	–	Yes	0.29	0.00
		Placebo		−34.52		–	37.45	–		0.40	0.00
Rechberger 2018 [32] (Hysterectomy)^B,D^	215	MIV	Yes	−19.47	Yes	15.90	31.80	–	Yes	0.00	0.00
		Morphine		0.77		28.80	–	–		0.10	0.00
		Placebo		4.61		48.00	96.00	–		0.00	0.00
Reinhart 2000 [56] (Bunionectomy)	38	Ketorolac	Yes	2.86	No	–	–	–	No	–	–
		Placebo ^C^		4.87		–	–	–		–	–
Rindos 2019 [57] (Hysterectomy)	183	Acetaminophen	Yes	2.38	No	–	–	–	No	–	–
		Placebo		2.86		–	–	–		–	–
Singla 2018 [34] (Abdominal)^B^	219	MIV	Yes	−1.1	Yes	18.30	26.90	–	Yes	0.35	0.04
		Placebo		−0.91		21.90	35.35	–		0.51	0.02
Singla 2010 [58] (Orthopedic)	185	Ibuprofen	No	–	Yes	41.10	–	–	Yes	0.27	–
		Placebo		–		59.50	–	–		0.19	–
Takeda 2019 [59] (Orthopedic)	97	Acetaminophen	No	–	Yes	80.01	–	–	No	–	–
		Placebo		–		81.31	–	–		–	–
Thybo 2019 [60] (Orthopedic)	281	Acetaminophen	No	–	Yes	36.00	–	–	Yes	0.04	0.03
		Ibuprofen		–		26.00	–	–		0.01	0.04
Wilson 2018 [61] (Cesarean)	141	Acetaminophen	No	–	Yes	20.00	47.00	–	No	–	–
		Placebo		–		32.00	48.00	–		–	–
Wong 2010 [62] (Abdominal)	66	Parecoxib	No	–	Yes	26.00	–	43.50	Yes	0.00	–
		Ketorolac		–		29.40	–	55.50		0.12	–

Abbreviations: *RCT* Randomized Clinical Trial, *MME* Morphine milligram equivalent, *ORADE* Opioid-related adverse drug events, *SPID* Sum of pain intensity differences, *GI* Gastrointestinal

^a^Assumes diclofenac suppositories are common practice for pain control in Cesarean sections

^b^Uses the 2-h windowed last observation carried forward (W2LOCF) value

^c^Used group KIV versus group L from the original report

^d^MME values at week 48 were extrapolated from MME values in the given study reported before hour 48 based on a regression using data from the other reatined studies for the relationships betwwen time and MME utilization

^e^MME at 48 weeks for was calculated from the median or median oxycodone use, which was converted to MMEs using a conversion factor of 1.4 per the guidance from the Centers for Medicare and Medicaid Services

### Outcomes from NMA

#### Pain

**Fig. 3:**
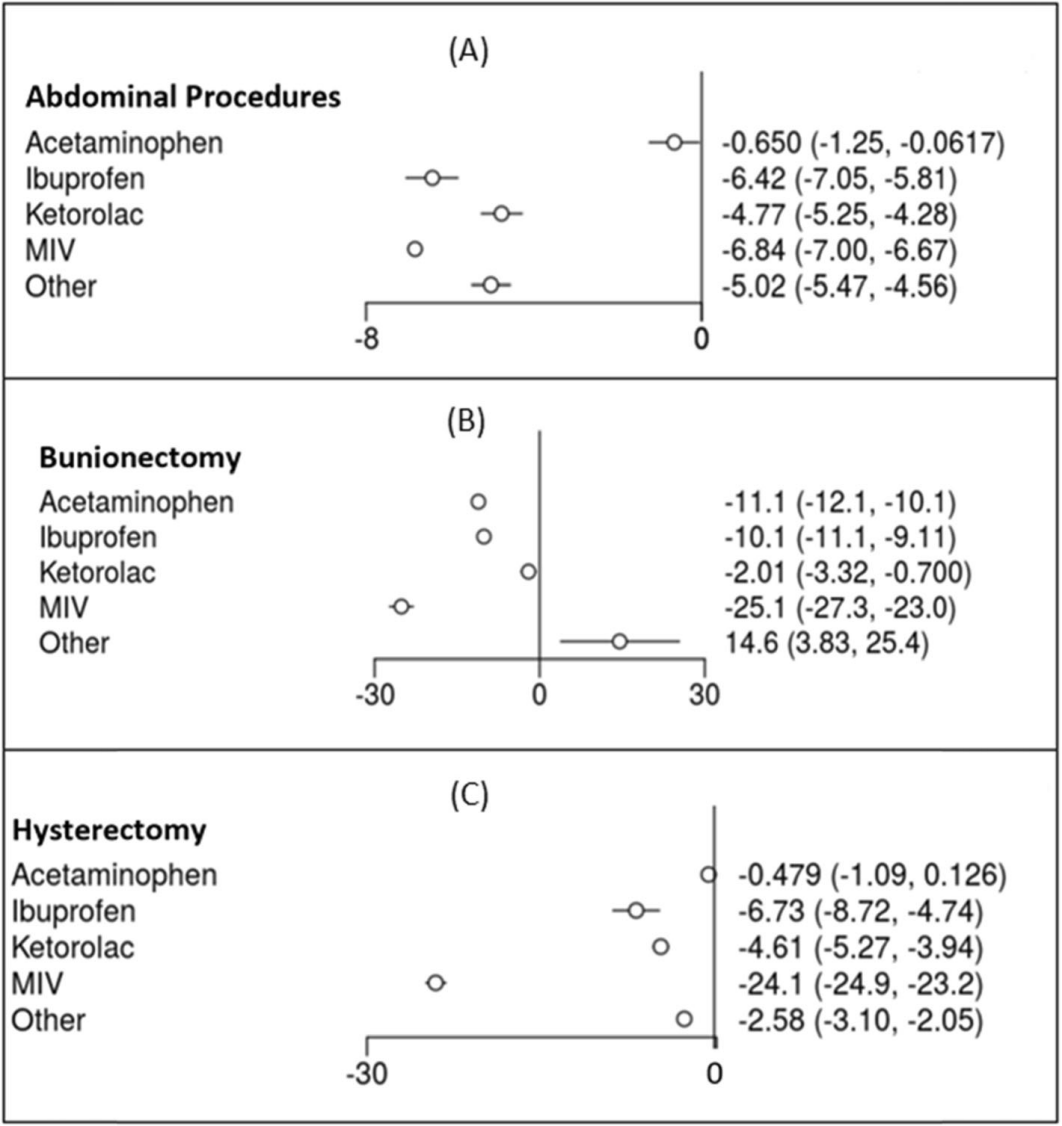
Summed pain intensity difference up to postoperative hour 24 (SPID 0–24) **a** Abdominal procedures **b** Bunionectomy **c** Hysterectomy

A total of sixteen studies contributed to the analysis for pain for abdominal, bunionectomy, and hysterectomy procedure categories: Orthopedic procedures were excluded for the pain outcome category because pain outcomes were not reported for MIV for this category. Among abdominal procedures, MIV was associated with significantly greater pain reductions versus acetaminophen, ketorolac, other medications, and placebo (Fig. 3a). MIV was nominally more effective in reducing pain versus ibuprofen, but the confidence intervals overlapped (Fig. 3a). However, the SUCRA ranking table indicated an 89.6% probability that MIV was the most effective treatment for abdominal procedures versus a 10.4% probability for ibuprofen (Table 2).

**Table 2:** Treatment Ranks Pooled Across Procedures for SPID 24 (Hours 0–24)^*a*^

Rank	Abdominal ^b^	Bunionectomy	Hysterectomy ^*b*^	Orthopedic
1	MIV (89.6%)	MIV (100%)	MIV (99.8%)	Results not included here because pain scores for MIV patients who underwent orthopedic procedures were not reported.
2	Ibuprofen	Acetaminophen	Ibuprofen	
3	Other	Ibuprofen	Other	
4	Ketorolac	Other	Ketorolac	
5	Acetaminophen	Ketorolac	Acetaminophen	
6	Placebo	Placebo	Placebo	

Note: Probabilities of top ranking are given in parentheses

Abbreviations: *MIV* Investigational IV meloxicam 30 mg

^*a.*^ The 24-h period was the longest common follow-up time among procedure categories

^*b.*^ Abdominal procedures and hysterectomy were included only open procedures

For bunionectomy, MIV was significantly more effective for pain reduction versus all other treatment options (as represented by SPID 24). As indicated in the forest plot (Fig. 3b) and the SUCRA ranking table (Table 2), the order of treatments with respect to efficacy for the pain outcome (SPID 24, best to worst) was MIV, acetaminophen, ibuprofen, ketorolac, placebo, and other. In the hysterectomy procedure category, MIV was again the most effective treatment option for reducing pain for up to 24 h postoperatively (Fig. 3c, Table 2). The order of treatments with respect to efficacy for the pain outcome (SPID 24, best to worst) was MIV, ibuprofen, ketorolac, other, acetaminophen, and placebo.

#### Morphine milligram equivalents

**Fig. 4:**
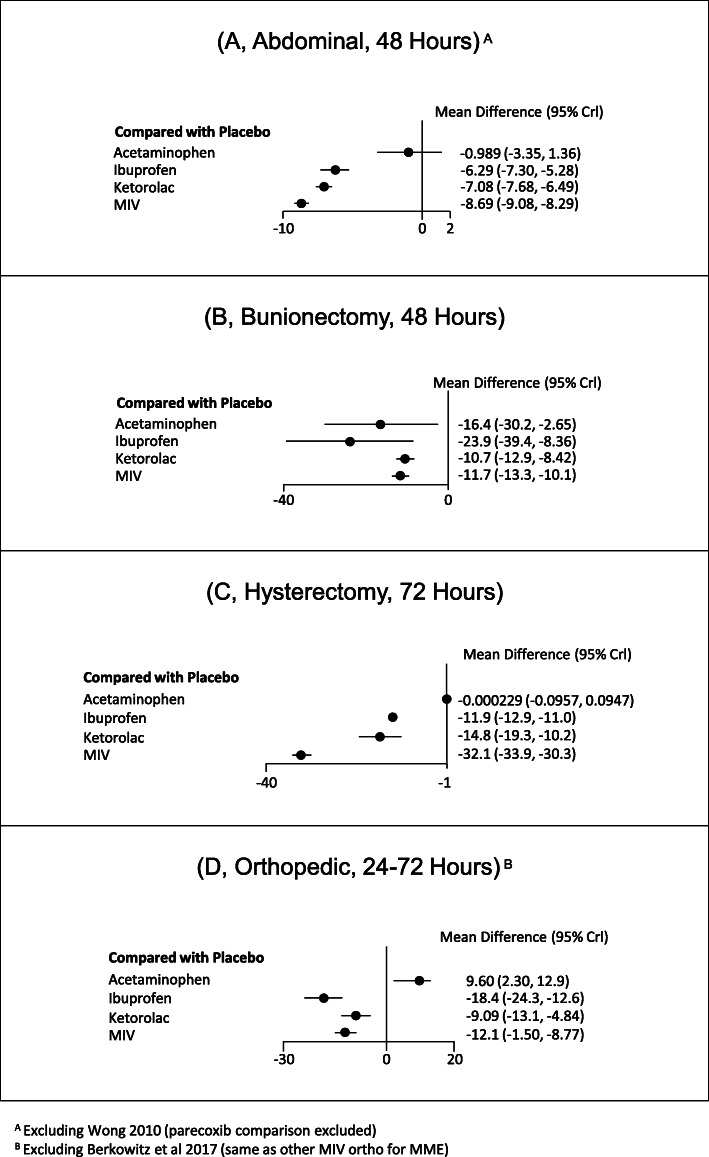
Percent difference in MME reduction for all procedures

Seventeen studies contributed to the analysis for MME across all procedures. Overall, MIV was associated with significant reduction in MME  in all procedure categories (Fig. 4) versus placebo. For abdominal procedures (Fig. 4a), MIV was associated with a  38% higher, significant reduction (− 8.7 [− 9.1, − 8.3] vs -6.3 [-7.3, -5.3]) in MME used for rescue treatment up to 48 h postoperatively compared with ibuprofen, a 23% higher, significant reduction (− 8.7 [− 9.1, − 8.3] vs -7.1 [-7.7, -6.5]) compared with ketorolac, and a 778% higher, significant reduction (-8.7 [-9.1, -8.3] vs -1.0 [-3.3, 1.4]) versus acetaminophen. For bunionectomy, the treatment options were statistically equivalent (Fig. 4b). For hysterectomy, MIV was associated with a >1,000%  higher significant reduction (−32.1 [−33.9, − 30.3%] vs - <0.1 [-0.1, 0.1]) in MME used for rescue treatment up to 48 h postoperatively compared with acetaminophen and 117% higher significant reduction (−32.1 [−33.9, −30.3] vs -14.8 [-19.3 vs -10.2]) compared with ketorolac. MIV was also associated with a 170% higher significant reduction (−32.1 [−33.9, -30.3] vs -11.9 [-12.9, -11.0]) in MME used for rescue treatment up to 48 h compared with ibuprofen (Fig. 4c). Finally, for orthopedic procedures, MIV was associated with a significant reduction (−12.1 [−15.0 , −8.8] vs 9.6 [2.3, 12.9]) in MMEs used for rescue treatment up to 48 h postoperatively compared with acetaminophen. No significant results were found for ketorolac or ibuprofen (Fig. 4).

#### ORADE

**Fig. 5:**
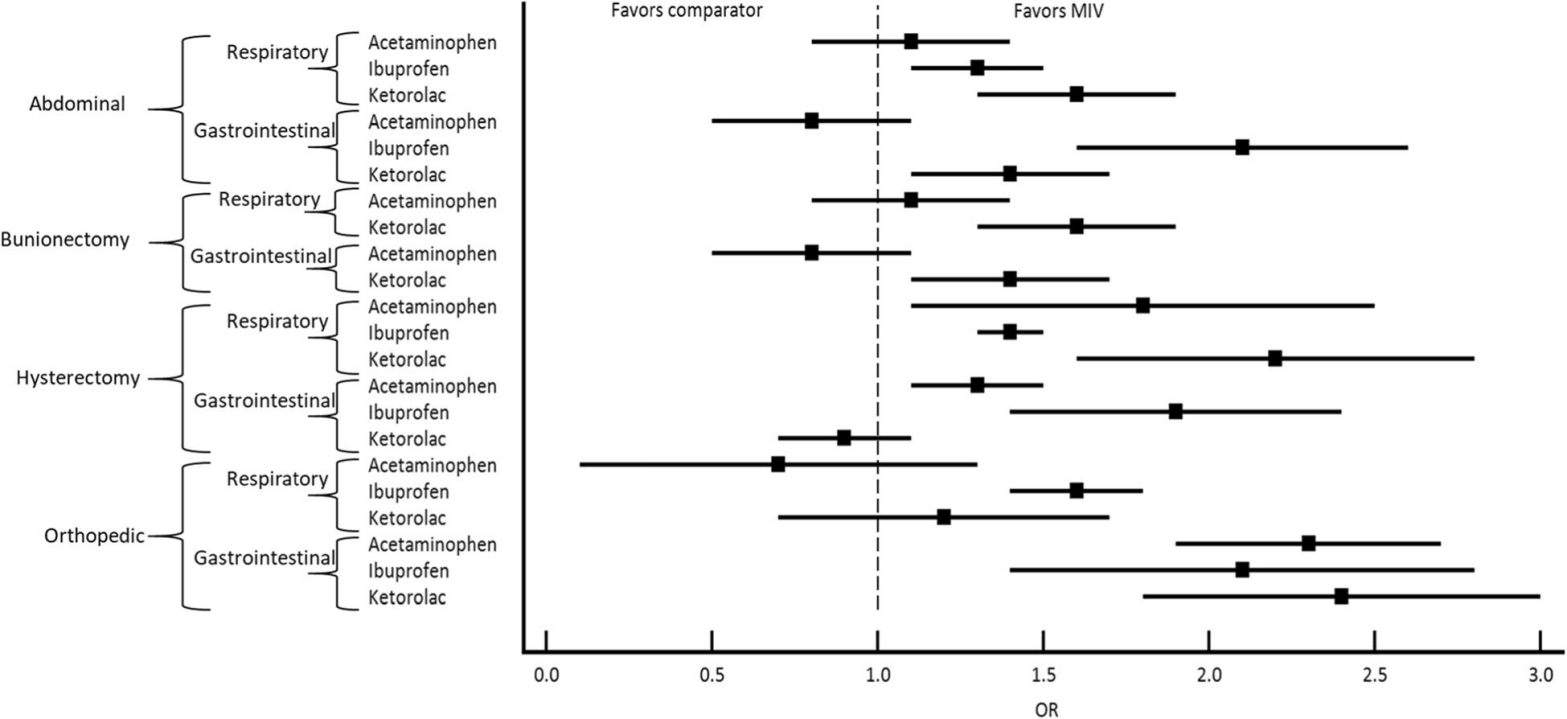
ORADE outcomes for all procedures by postoperative hour 48

Overall, the odds of ORADE were lower with MIV than with the other comparators (including all comparisons regardless of postoperative pain threshold; additional data available upon request). Across the abdominal procedure category, the odds of having either respiratory or gastrointestinal ORADE for Hours 0–48 associated with both ibuprofen and ketorolac were significantly higher compared with MIV (OR, Random Effects [95% CrI]: Ibuprofen (Respiratory): 1.3 [1.1, 1.5]; Ibuprofen (Gastrointestinal): 2.1 [1.6, 2.6]; Ketorolac (Respiratory): 1.6 [1.3, 1.9]; Ketorolac (Gastrointestinal): 1.4 [1.1, 1.7]). For bunionectomy, the odds of having both respiratory (OR, Random Effects [95% CrI]: 1.6 [1.3, 1.9]) or gastrointestinal (OR, Random Effects [95% CrI]: 1.4 [1.1, 1.7]) ORADE for Hours 0–48 associated with ketorolac were significantly higher compared with MIV. The findings, however, were not significant for acetaminophen compared with MIV (OR, Random Effects [95% CrI], Respiratory: 1.1 [0.8, 1.4]; Gastrointestinal: 0.8 [0.5, 1.1]). For hysterectomy, the odds of having both respiratory or gastrointestinal ORADE for Hours 0–48 associated with both ibuprofen and acetaminophen were significantly higher compared with MIV (OR, Random Effects [95% CrI]: acetaminophen (Respiratory): 1.8 [1.1, 2.5]; acetaminophen (Gastrointestinal): 1.3 [1.1, 1.5]; Ibuprofen (Respiratory): 1.4 [1.3, 1.5]; Ibuprofen (Gastrointestinal): 1.9 [1.4, 2.4]) (Fig. 3c). Significantly higher odds of respiratory ORADE (OR, Random Effects [95% CrI]: 2.2 [1.6,2.8] were found to be associated with ketorolac in comparison with MIV with no significant finding for gastrointestinal ORADE (OR, Random Effects [95% CrI]: 0.9 [0.7,1.1]. For miscellaneous orthopedic procedures, the odds of having a respiratory or gastrointestinal ORADE for Hours 0–48 associated with both ibuprofen and ketorolac were significantly higher compared with MIV except for ketorolac (respiratory), (OR, Random Effects [95% CrI]: Ibuprofen (Respiratory): 1.6 [1.4, 1.8]; Ibuprofen (Gastrointestinal): 2.1 [1.4, 2.8]; Ketorolac (Respiratory): 1.2 [0.7, 1.7]; Ketorolac (Gastrointestinal): 2.4 [1.8, 3.0]) (Fig. 5).

## Discussion

Meloxicam, a NSAID, was first approved in the US in 2000 for oral use [28]. Oral meloxicam has been marketed to treat symptoms of osteoarthritis and rheumatoid arthritis but has a slow onset of action largely caused by its poor water solubility [63]. Intravenous meloxicam is an approved product that utilizes the NanoCrystal™ platform, a technology designed to enable enhanced bioavailability of poorly water-soluble drug compounds [63]. Based on the results of multiple clinical trials, MIV has been found to provide relief of moderate to severe acute pain, alone or in combination with other analgesics within the first 15 min after dosing and up to 24 h after dosing compared to placebo. The current study assessed the safety and efficacy of MIV relative to other IV non-opioid analgesics for managing moderate-severe postoperative pain by conducting an NMA. The outcomes associated with pain, MME and ORADEs (respiratory and gastrointestinal) were indirectly compared with non-opioid IV analgesics comparators including acetaminophen, ibuprofen and ketorolac.

In reducing pain intensity (SPID 24), MIV was significantly more effective than all comparators for all procedure categories. In the case of MME, similar findings were observed, where MIV was associated with significant reduction in MME for most comparisons at 48- and 72-h postoperatively. Among MME comparisons for abdominal procedures and hysterectomy, a significant reduction in MME was observed with MIV compared to all comparators (acetaminophen, ibuprofen, and ketorolac). Mixed results were observed among other procedure categories, wherein, MIV was equivalent to other comparators in bunionectomy and orthopedic procedures, except versus acetminophen where a significant reduction in MME was observed with MIV in comparison. MIV was also observed to be associated with lower odds of ORADEs compared to other NSAIDs and acetaminophen for most procedures and comparators. Compared with ketorolac, MIV resulted in lower odds of ORADE for all procedures except hysterectomy (gastrointestinal ORADE) and orthopedic (respiratory ORADE) procedures. In case of acetaminophen, MIV did not show a reduction in ORADEs for most procedure categories including abdominoplasty (respiratory and gastrointestinal), bunionectomy (respiratory and gastrointestinal) and orthopedic (respiratory) except for hysterectomy and orthopedic (gastrointestinal).

This study has several limitations. First, the outcomes evaluated in this study were not controlled for dose-dependent effects as there were not enough number of studies to stratify by doses and consider variable dosing. However, this could have impacted outcomes if in some studies there were treatment groups with extremely higher/lower dosing, which was not found in our case. The doses were found to be within a narrow range. Secondly, outcomes are highly sensitive to underlying assumptions. For example, assumptions regarding what constituted a similar procedure to abdominoplasty affected which studies were chosen for that comparison. Since these were not abdominoplasties, there is some uncertainty regarding the external validity of the comparison. Also, confidence intervals for continuous data extracted from the literature were imputed when not reported in the original reports. This imputation requires assumptions regarding the shape of the probability distribution for those confidence intervals, which in turn affects the confidence intervals around the outcomes we produced. Third, some trials were not powered for evaluating the extracted outcomes. Fourth, few studies in the postoperative pain literature reported moderate-severe pain after surgery as inclusion criterion. Since the focus of the study was to compare (indirectly) MIV with other non-opioid analgesics, it was important that we included studies that had similar inclusion and exclusion criteria as MIV studies. Given that only limited number of current studies had the same inclusion criteria as MIV studies, relatively older studies were included in analysis to maintain homogeneity within the selected studies. Fifth, MIV trials were unique due to the two-hour window analysis of pain. Sixth, comparisons were not controlled for timing of pain assessment relative to rescue administration.

## Conclusion

The current study found that among patients reporting moderate to severe postoperative pain MIV was superior in pain reduction for abdominoplasty, bunionectomy and hysterectomy when compared with acetaminophen, ibuprofen, and ketorolac. In reducing MME, MIV was superior or equivalent to all comparators and among all procedure categories except ibuprofen (bunionectomy and hysterectomy) and ketorolac (bunionectomy and orthopedic). Finally, MIV reduced the odds of ORADEs in most comparisons except ketorolac for hysterectomy (gastrointestinal ORADE) and orthopedic (respiratory ORADE) procedures, and acetaminophen for abdominoplasty (respiratory and gastrointestinal), bunionectomy (respiratory and gastrointestinal) and orthopedic (respiratory). Results should be interpreted with caution due to the indirect nature of the comparisons to approved IV non-opioid analgesics. Nevertheless, these results suggest MIV 30 mg may provide better pain reduction with similar or better safety to approved IV non-opioid analgesics.

## Data Availability

The data used and/or analyzed during the current study are available from the corresponding author on reasonable request.

## Abbreviations

CrI: Credible interval
COX-1 and/or COX-2: Cyclooxygenase
MIV: IV meloxicam 30 mg
MME: Morphine milligram equivalent
NMA: Network-meta-analysis
NSAID: Non-steroidal anti-inflammatory drug
OR: Odds ratio
ORADE: Opioid-related adverse drug event
PICOT: Population, interventions, comparators, outcomes, time period
RCT: Randomized controlled clinical trial
SMD: Standardized mean difference
SPID: Sum of pain intensity difference
SUCRA: Surface under the cumulative ranking curve
